# Acceptability of woman-delivered HIV self-testing to the male partner, and additional interventions: a qualitative study of antenatal care participants in Malawi

**DOI:** 10.7448/IAS.20.1.21610

**Published:** 2017-06-26

**Authors:** Augustine Talumba Choko, Moses Kelly Kumwenda, Cheryl Case Johnson, Doreen Wongera Sakala, Maria Chifuniro Chikalipo, Katherine Fielding, Jeremiah Chikovore, Nicola Desmond, Elizabeth Lucy Corbett

**Affiliations:** ^a^ TB/HIV Theme, Malawi Liverpool Wellcome Trust Clinical Research Programme, Blantyre, Malawi; ^b^ College of Medicine, University of Malawi, Blantyre, Malawi; ^c^ Infectious Disease Epidemiology, London School of Hygiene & Tropical Medicine, London, UK; ^d^ Department of Clinical Research, London School of Hygiene & Tropical Medicine, London, UK; ^e^ HIV/AIDS, Sexually Transmitted Infections & TB, Human Sciences Research Council, Durban, South Africa; ^f^ Department of Clinical Research, Liverpool School of Tropical Medicine, Liverpool, UK; ^g^ University of Malawi, Kamuzu College of Nursing, Blantyre, Malawi; ^h^ Department of HIV, World Health Organization, Geneva, Switzerland

**Keywords:** HIV, rapid HIV test, HIV self-testing, financial incentives, ANC, Malawi, sub-Saharan Africa

## Abstract

**Introduction**: In the era of ambitious HIV targets, novel HIV testing models are required for hard-to-reach groups such as men, who remain underserved by existing services. Pregnancy presents a unique opportunity for partners to test for HIV, as many pregnant women will attend antenatal care (ANC). We describe the views of pregnant women and their male partners on HIV self-test kits that are woman-delivered, alone or with an additional intervention.

**Methods**: A formative qualitative study to inform the design of a multi-arm multi-stage cluster-randomized trial, comprised of six focus group discussions and 20 in-depth interviews, was conducted. ANC attendees were purposively sampled on the day of initial clinic visit, while men were recruited after obtaining their contact information from their female partners. Data were analysed using content analysis, and our interpretation is hypothetical as participants were not offered self-test kits.

**Results**: Providing HIV self-test kits to pregnant women to deliver to their male partners was highly acceptable to both women and men. Men preferred this approach compared with standard facility-based testing, as self-testing fits into their lifestyles which were characterized by extreme day-to-day economic pressures, including the need to raise money for food for their household daily. Men and women emphasized the need for careful communication before and after collection of the self-test kits in order to minimize the potential for intimate partner violence although physical violence was perceived as less likely to occur. Most men stated a preference to first self-test alone, followed by testing as a couple. Regarding interventions for optimizing linkage following self-testing, both men and women felt that a fixed financial incentive of approximately USD$2 would increase linkage. However, there were concerns that financial incentives of greater value may lead to multiple pregnancies and lack of child spacing. In this low-income setting, a lottery incentive was considered overly disappointing for those who receive nothing. Phone call reminders were preferred to short messaging service.

**Conclusions**: Woman-delivered HIV self-testing through ANC was acceptable to pregnant women and their male partners. Feedback on additional linkage enablers will be used to alter pre-planned trial arms.

## Introduction

Up to 54% of people with HIV in sub-Saharan Africa (SSA), the region hardest hit by the HIV epidemic, do not know their status [1]. The mortality rate in men is nearly twice that of women within the first three months of starting antiretroviral therapy (ART) [2], predominantly due to late diagnosis [3]. Although HIV prevalence is higher in women than in men in most parts of Africa [4], men account for a disproportionately high number of undiagnosed HIV infections [5]. Indeed, despite rapid scale up of HIV testing services (HTS) in SSA, high levels of discrimination and stigmatization associated with HIV testing and positive diagnosis impede impactful progress [6]. A number of strategies have shown effect on increasing the uptake of HIV testing for both men and women including home-based testing, provider-initiated testing and counselling, and the promotion of couples-testing during antenatal visits [7,8]. A novel alternative strategy, HIV self-testing (HIVST), is now the subject of wider-scale implementation, with ongoing research aiming to define best practice for various populations [9].

Antenatal care (ANC) services provide a unique opportunity for reaching partners using couple-centred interventions including couples HIV testing services (cHTS) [10]. Male involvement may improve maternal outcomes, in addition to contributing to more effective implementation of prevention of mother to child transmission of HIV [11]. Male partner tracing in Malawi [12] and distribution of woman-delivered HIVST during ANC attendance in Kenya [13,14] were shown to be effective ways to increase uptake of HTS among men in three recent studies.

HIVST which involves an individual collecting their own sample, conducting the test and interpreting their result [15] overcomes most traditional barriers which hinder people’s access to HTS. In particular, men commonly cite lack of transport, opportunity cost, being busy, fear of testing positive while with partner, and stigma among chief barriers to male partner testing and cHTS [16–18]. HIVST is usually offered and performed closer to people’s homes making it convenient and less costly to individuals [19,20]. A self-test guarantees privacy and confidentiality which may allay fears of testing positive with a sexual partner, remove the stigma of being in the vicinity of an HIV testing point [21] and caters for individuals whose busy schedule otherwise makes testing less convenient [15]. Therefore, it seems little surprise that HIVST has achieved high uptake in different populations across the world [22,23]. As part of preliminary work to inform interventions for a proposed multi-arm multi-stage cluster-randomized trial, we sought views regarding the acceptability of offering HIV self-test kits alone or in combination with a linkage intervention to ANC attendees aimed at their male partners.

## Methods

### Design, setting and participants

**Figure 1.**
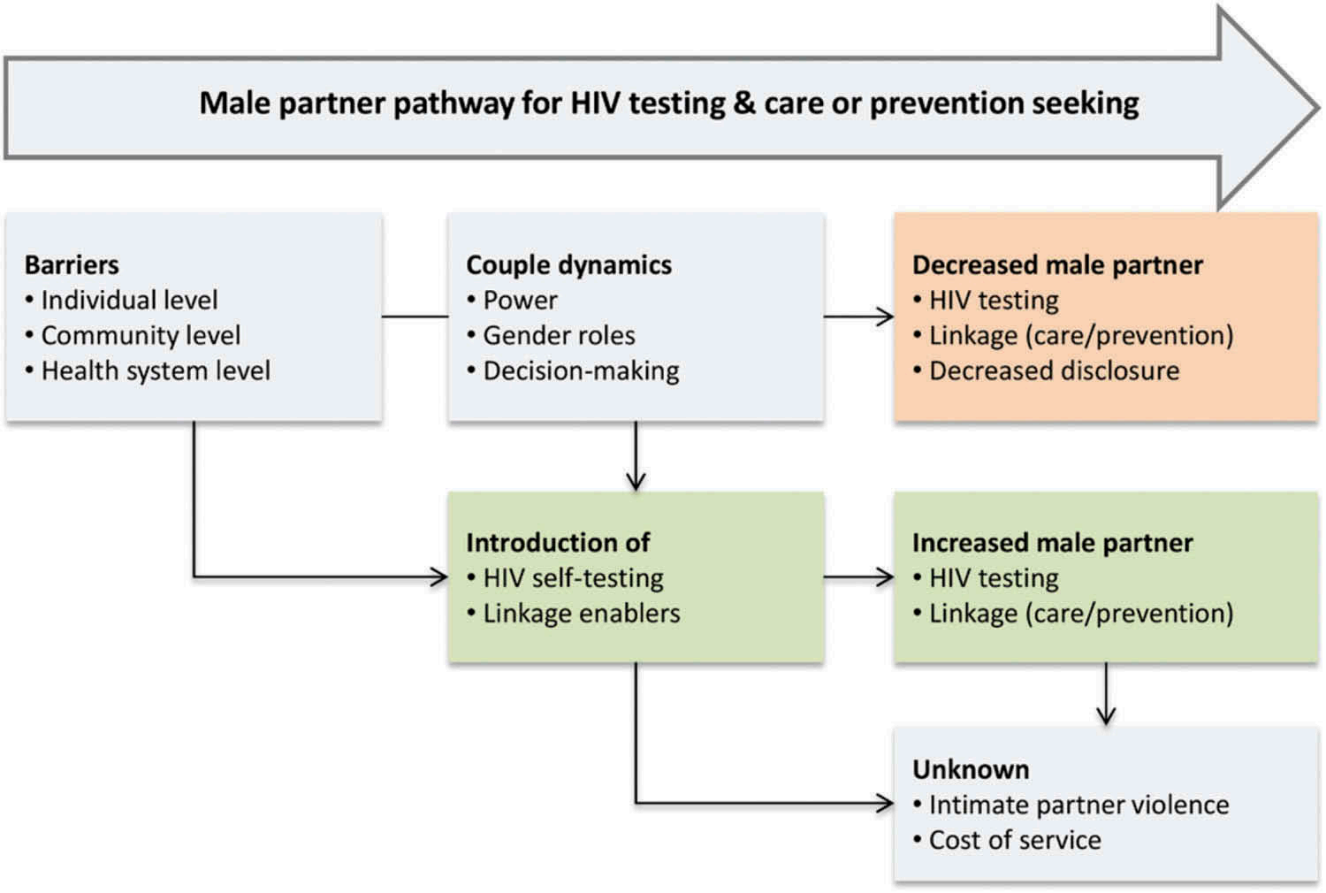
**Conceptual framework**.

We carried out a formative qualitative study using focus group discussions (FGDs) and in-depth interviews (IDIs) for data collection between October 2015 and February 2016. The study recruited ANC attendees at three primary health clinics (PHCs) of Ndirande, Bangwe and Zingwangwa, in urban Blantyre, Malawi. These three PHCs were chosen because they serve a low-income urban population that is likely to benefit from future scale-up of the interventions under investigation. Male partners of pregnant women, not necessarily as couples, who were attending these PHCs were also invited to participate in the study. Our conceptual framework (Figure 1) is based on the fact that there are many barriers which hinder or delay male partners from testing for HIV and linking to care or prevention. We expected that pregnant women-delivered HIVST alone or in combination with another intervention would address some of these barriers thereby increasing male partner testing and linkage into care or prevention.

### Sampling, sample size and eligibility

Participants in the FGDs were purposively sampled with the aim of having between 8 and 12 participants per FGD. Two FGDs each with women only, men only and mixed gender were conducted (6 in total), followed by 20 IDIs (10 men and 10 women) with some of the FGD participants. A group information session was held with women in the ANC waiting area; those who were interested were then screened for eligibility after completing their ANC attendance processes. Inclusion criteria were age (≥18 years), residence in urban Blantyre, current ANC attendance (for women) or having a pregnant partner currently attending ANC (for men), and willingness to provide consent.

Female FGD participants were randomly selected from the list of eligible ANC attendees. For male FGD participants, we asked for partner contact information from the eligible ANC attendees, contacted the male partners to ascertain their eligibility and willingness to participate, and randomly selected (22–25) for men-only FGD and seven for the mixed gender FGD. At the end of each FGD, participants were asked to participate in a follow-up IDI. Fifteen men and 15 women were randomly selected from among those willing to participate in the IDIs (Table 1). All interviews were conducted at a convenient location and time in consultation with participants. A demonstration of HIVST was made during FGDs but participants were not offered the test, hence all results are based on a hypothetical framework.

**Table 1.** Summary of participation

	Approached	Accepted		Participated	
Data source	*n*	*n*	%	*n*	%
FGD 1, women only	16	14	87.5	8	57.1
FGD 2, women only	19	15	78.9	6	40.0
FGD 3, men only	25	10	40.0	8	80.0
FGD 4, men only	22	7	32.0	6	85.7
FGD 5, mixed gender	14	6	42.9	6	100
FGD 6, mixed gender	12	8	66.7	8	100
Total FGDs	108	60	55.6	42	70.0
IDI, men	15	13	86.7	10	76.9
IDI, women	15	15	100	10	66.7
Total IDIs	30	28	93.3	20	71.0
FGD: focus group discussion; IDI: in-depth interview.					

Open-ended questions in the local language seeking to capture different key issues such as barriers to male partner testing, HIVST, adverse events due to HIVST and additional interventions were used to guide the FGDs (Figure 1). Similar themes were covered in IDIs although with a greater focus on soliciting individual perspectives. Doing the IDIs after participating in FGD likely allowed participants to reflect more on their individual experiences. Furthermore, IDIs may have helped participants feel more at ease to express their views than was the case in the FGD [24].

### Ethical considerations

The study was approved by the University of Malawi, College of Medicine Research and Ethics Committee (COMREC), approval number: P.08/15/1784 and London School of Hygiene & Tropical Medicine Ethics Committee, approval number: 10332. All participants either gave written informed consent in the local language, or witnessed consent plus thumbprint if illiterate before participation. An impartial witness, usually a nurse at ANC, read and helped the participants to understand the study information before consent was given. Participants were given MWK1000 (USD1.33) compensation for their time which is the minimum recommended by COMREC. Participants were given a number with which they were identified during FGDs, IDIs and during data analysis in order to uphold anonymity.

### Data analysis

FGDs and IDIs lasted an average of two hours and 40 min, respectively. Data were captured using digital audio recorders and field summaries; the latter provided quick impressions about emerging issues and helped researchers to determine whether or not saturation of information was reached. Recorded data were translated and transcribed verbatim before being cleaned and stripped of any details that might make it possible to identify participants. Each transcript was manually coded by AC, MK, DS and MC using a predefined codebook and then compared across the four coders to assess inter-coder reliability. Simple descriptive content analysis was used to analyse the data in a process that involved the four researchers independently extracting data and later discussing each emerging category. The primary data coding was deductively done under two major themes namely: acceptability of woman-delivered HIVST and potential linkage enablers that could be added to woman-delivered HIVST.

## Results

### Participation and characteristics of participants

Acceptance to participate was higher for women (78.9–87.5%) compared to men (32–40%) for separate gender FGDs (Table 1). However, men were more likely to actually participate in FGDs or IDIs once they accepted the invitation (Table 1). Of the 42 FGD participants, 18 (42.9%) were men and 24 (57.1%) were women, with each FGD varying in size from 6 to 8 participants (Table 2). Men were older (median age: 28.5, inter quartile range (IQR): 25.0–31.0 vs. 23.5; IQR: 19.0–29.0), had higher educational attainment and were more likely to be employed compared to women. About two-thirds said they had ever tested together for HIV. Ten women and 10 men participated in IDIs as planned. We present the data under three key themes that emerged: barriers to couple testing at ANC; acceptability of woman-delivered HIVST; and additional interventions to HIVST to encourage partner linkage following HIVST. These themes echoed our conceptual framework (Figure 1).

**Table 2.** Characteristics of focus group discussion participants by gender (*N* = 42)

		Men		Women		Overall	
Characteristic		*n*	%	*n*	%	*n*	%
Total	Number	18	42.9	24	57.1	42	100
Age	Median (IQR)	28.5	25–31	23.5	19–29	27.5	22–30
PHC	Ndirande	8	44.4	10	55.6	18	42.9
	Zingwangwa	8	50.0	8	50.0	16	38.1
	Bangwe	2	25.0	6	75.0	8	19.0
Education	Primary	6	30.0	14	70.0	20	47.6
	Secondary	7	50.0	7	50.0	14	33.3
	Higher	5	62.5	3	37.5	8	19.1
Occupation	Paid employee	6	100	0	0.0	6	14.3
	Paid domestic worker	3	100	0	0.0	3	7.1
	Self-employed	6	66.7	3	33.3	9	21.4
	Unemployed	1	4.8	20	95.2	21	50.0
	Student	1	100	0	0.0	1	2.4
	Other	1	50.0	1	50.0	2	4.8
Marital status	Married	17	44.7	21	55.3	38	90.5
	Never married	1	33.3	2	66.7	3	7.1
	Separated	0	0.0	1	100	1	2.4
Tested with partner before?	Yes	13	48.2	14	51.8	27	64.3
	No	5	33.3	10	66.7	15	35.7
IQR: inter quartile range; PHC: primary health clinic.							

### Barriers to couple testing at ANC

Participants in both FGDs and IDIs cited well-known and documented barriers operating at different levels (structural, community, couple, individual and economic) as hindrances to couple testing at ANC. Of note, stigma, discrimination and costs, both direct and indirect as well as time constraints in light of competing needs featured highly in discussions and interviews:

Considering what happens here at ANC clinic, I don’t see my husband escorting me anymore because you find he is alone among many women and he has to listen to some things concerning birth. … it’s better if couples are given a private room to discuss. (Female, FGD, Ndirande)

Fear of stigma and perceived lack of confidentiality was of concern to men:

Before I escorted my wife to antenatal clinic I thought, if I go I will be the only man in a group of women and if I am tested all these women will know my status. (Male, Zingwangwa, FGD)

Sociocultural norms often shaped men’s decision-making around ANC and couple testing:

My friends were just laughing at me, for example the day before yesterday when my wife told me to come to ANC clinic, when I told my friends they all said “I was stupid because ANC clinic is not for men”. (Male, Ndirande, FGD)

Couple-related aspects such as fear of blame, divorce and partner’s reaction were gendered and were raised mostly by men especially in the context of potential discordant results:

The problem is that you are afraid and worried about couple testing because you think if one tests negative and another positive it means the marriage will break up. Because of this fear and worry you prefer not to test. (Male, Ndirande, FGD)

Men often said they could not balance economic needs and ANC attendance particularly as they had to get income on a daily basis:

A person leaves his economic activities for the whole day to go to the clinic. Here in town, very few people have food for tomorrow. Most of us only have food for today. Now you go to the clinic, when you return, people (*at home*) have no food, so it’s better to go look for food f than to the ANC clinic. (Male, FGD, Zingwangwa)

### Acceptability of pregnant woman-delivered HIVST

Most participants felt that this model of delivering HIVST would address most of the barriers that deter men from testing together with their partners at ANC. Convenience was among the factors men highlighted.

I feel like it’s acceptable because maybe the day that the woman wants to go to ANC clinic you might not be able to escort her, so she can just bring you the test kits when she is coming back from ANC and the next time she is going for ANC then you can go together. (Male, FGD, Ndirande)

Men and women felt that this model offers privacy and would remove stigma associated with men attending ANC and testing:

Actually it will be very simple because it will be like “ohh they have helped me here, I don’t have to go to antenatal care and meet a lot of women.” It is better I just do it on my own because you cannot be shy with your wife especially if you know that your ways are faithful, you can just do it right there with your wife. (Male, Mixed FGD, Ndirande)

For men, being the first to know meant HIVST offered control over their testing environment including disclosure, unlike the standard testing procedure:

Men would definitely accept … they would say, “aaah, why should the doctor test me? Aaah, it’s better to be the first to know my HIV status.” You would feel shy when meeting the doctor who knows that you are HIV positive. (Male, IDI, Ndirande)

Men emphasized the need to be the first to know your results:

It [*being tested at ANC*] did not really have privacy, but with how you have set it up to say one can test themselves using the test kits and know the results by themselves first then the privacy is there. (Male, FGD, Ndirande)

#### Concerns around woman-delivered HIVST

Some participants also highlighted *lack of immediate counselling* as potentially problematic as well as concerns around *trust*. Some men and women indicated that physical or psychological intimate partner violence (IPV) or verbal abuse may occur depending on how the woman has presented the issue or if prior consultation was not done with the man:

There are some women with poor approach. They just begin by saying here are the test kits you have been dodging the subject and today I have brought them and we will test here at home […*laughs*]. So you can just slap her […*laughs*] and say go tell your Doctor to self-test with you not me […*laughs*]. (Male, FGD, Zingwangwa)

They [*taking self-test kits for him*] can cause misunderstandings if the man doesn’t like it. He can ridicule you in everything you do or say. He will say you are stupid because you make decisions on your own and this can cause arguments and you can have no peace of mind. (Female, FGD, Zingwangwa)

Having said this, there was a general perception that a pregnant woman may not suffer physical IPV as a consequence of delivering HIVST to the partner:

Even if the man can get angry, it’s difficult for a man to beat his pregnant wife no matter how short tempered he is – even if he has a history of beating her or abusing her in other ways. (Male, FGD, Ndirande)

Women also agreed that physical IPV may be less likely to occur solely by dislike of test kits:

…because in marriages there are things that make one to fear. Other men are difficult, yes others would manage but some cannot manage … The only problem would perhaps be that he would just refuse and that is it, but not reaching the extent of beating [*the woman*], no. (Woman, FGD, Zingwangwa)

There were concerns from participants regarding the potential lack of counselling for the male partner with the woman-delivered HIVST model considering that HIV remains greatly feared by the society:

AIDS is something scary, so if the person self-tests without a doctor maybe he can have suicidal thoughts or try to hurt himself. … When you are tested at the hospital, afterwards they counsel you. So if you just test yourself and find that you are positive, you can hurt yourself because there is no one to advise you about what to do next. (Male, FGD, Bangwe)

Some participants expressed concern that when a woman brings HIVST kits into the relationship, it might be construed as a sign of mistrust towards the male partner:

…you have brought me these? It just shows that you don’t trust me. So there really can be some problems, more especially if the approach was also not good. (Male, Mixed FGD, Ndirande)

Conversely, men were said to prefer using the test kits secretly first particularly if they knew they had engaged in infidelity:

…you just go out secretly and follow the method and right there it’s as easy as drinking a glass of water. You quickly place it in the bottle and hide it since you want to check yourself first (*participants laugh*). When the results are out you will check them and you will know the outcome yourself right? (Male, FGD, Ndirande)

#### Communication

How a woman communicated to the male partner before and after collecting the test kits was considered vital to the success of the model:

The self-testing approach can be accepted by men provided they are told first before the woman collects the test kits. They should discuss it first as a family, that there is an approach being provided at ANC. Only after reaching an agreement can the woman collect the test kits. (Male, IDI, Ndirande)

Men suggested that bedtime is the best for introducing such an issue to the relationship:

… if the woman is smart, when people have just had supper, she goes ‘aah let’s retire now’. The time you go to your bedroom that’s the time you can now start telling him to say “my husband, I went to the hospital and I have come back with these [*test kits*]… this is how we use these materials.” (Male, FGD, Zingwangwa)

The suggestion of using bedtime to introduce HIVST also featured highly in FGDs with women:

When he is coming, show that he is welcome home. Greet him and ask how work was then you can discuss the other things in the bedroom when going to bed. When he gets home, don’t just start talking to say I was at the hospital and this is what they have said and they have given me test kits so that we test ourselves. Is the man going to listen? But when we go to the bedroom where things between a man and a woman end. (Female, FGD, Ndirande).

Some participants suggested the use of an official letter addressed to the male partner from the clinic as a means of easing the burden on women to introduce such a sensitive issue:

We were given these forms [*the invitation letters*], so I feel like it would be a good way to also give them [*men*] something to read. After reading, they would be able to understand and when you get the kits, it will be an issue that they already know about. (Female, FGD, Zingwangwa)

### Views regarding additional interventions to HIVST to encourage partner linkage following HIVST

Participants were asked about different predefined types of strategies that could be added to HIVST provided through ANC in order to increase HIV testing and linkage into care (such as antiretroviral therapy (ART)) or prevention (condom use, counselling uptake and voluntary medical male circumcision). These strategies included: fixed financial incentives (low-amount transport equivalent ($3); higher amount to cover transport and opportunity cost ($15); lottery incentive ($3 equivalent) with a 10% chance of winning; and short messaging service (SMS) or phone reminders). The choice of $3 was guided by a recent study conducted in the same setting which found that people spent an average USD3.91 to access HIV testing [20]. In short, virtually all participants preferred cash as opposed to voucher incentives.

#### Transport-equivalent incentive

Participants said that providing a low-amount financial incentive would increase the number of male partners who would test and link into care or prevention. Such a strategy would remove a crucial economic barrier linked to transport costs as shown in the quote:

Because when he self-tests, if you tell him to go to the clinic to receive counselling, he would say he has no transport to go there. But if transport money is there, he won’t have any excuse. (Female, FGD, Ndirande).

#### High-amount financial incentive

Participants agreed that high financial incentives of about $10 would definitely improve linkage into care or prevention as this would compensate for opportunity cost as illustrated by the quote below:

When you come to the clinic, you spend the whole day with no food for today. Providing a high financial incentive would encourage other male partners, upon hearing that their friend just got food for the day by simply going to the clinic. (Male, FGD, Zingwangwa)

However, any amount considered excessive such as more than $10 was considered potentially problematic as it may lead to unintended negative consequences.

Some saw such an incentive as potentially promoting multiple sexual partnerships: or discouraging child spacing in marriages:

You can wish you had brought with you three pregnant wives [*for maximum financial incentive*]. (Male, FGD, Zingwangwa)

It will be difficult for people to have adequate child spacing [*with high financial incentive*]. Male, FGD, Ndirande

#### Lottery type of incentives

Views regarding lottery-based incentives were predominantly negative among both men and women as most participants perceived lotteries as being highly inequitable and unfair:

You may find that after the lottery, people that are better off – who came by car – are the ones who win the lottery. What would other people think?. (Male, FGD, Ndirande)

Other participants felt that a lottery might even, as a result, have an unintended negative group effect:

And its only one person who has won while the ones who have lost are many, so the message that will be spread will be from the ones that have lost because they are many. (Male, Mixed FGD, Ndirande)

However, some participants still felt that lotteries were acceptable to men and could help draw them into care, as described in the quote below:

It can also be good to the men because it will be like an encouragement for them to come and test; they will know that after testing “I may win a prize”. (Female, IDI, Ndirande)

#### SMS versus phone call reminder

Although participants agreed that SMS reminders would encourage male partners to test and link into care or prevention, they felt that a phone call reminder would offer greater room for dialogue and conversational engagement compared to an SMS reminder.

Not many people read the SMS when they receive it. … many just ignore it, mistaking it for Airtel [*Network provider*] promotional SMSs, even deleting before even reading. Better if it is phone calls…. (Male, FGD, Zingwangwa)

Problems that were highlighted with respect to phone call reminders mainly revolved around trust and lack of face-to-face dialogue when discussing sensitive issues. Women, for instance, expressed concern that their male partners may not like their phone numbers to be shared with the healthcare providers:

I feel like the approach of calling someone can cause marriage breakups… The man can wonder what sort of conversation culminated into his partner to give his phone number. (Female, Mixed FGD, Ndirande)

Being unable to read the mood of the call recipient and the mere absence of face-to-face interaction were flagged as potentially problematic with phone call reminders:

Other people maybe you call them when they are in a bad mood or they just quarrelled with the wife, that’s the problem of using phones. (Female, Mixed FGD, Ndirande)

## Discussion

Our investigation and data interpretation is based on a hypothetical framework in which women accessing ANC for their first visit would be offered HIV self-test kits alone or in combination with an additional intervention aiming to deliver to their male partner. This study has shown that providing HIVST kits to pregnant women who are accessing ANC with the aim of reaching out to their male partner would be acceptable to both men and women primarily because it was perceived to address key barriers associated with existing clinic-based HTS models. However, the results suggest that financial incentives may be useful in improving linkage into HIV care and prevention particularly in settings with extreme poverty. The use of lottery incentives was not preferred by participants, contrary to what is expected from economic theory which posits that given a high expected gain people are willing to take a risk [25]. The period of pregnancy is of high economic pressure and therefore may make partners risk averse. A number of modifications were made to the initial design of the cluster-randomized trial following results observed here (Table 3).

**Table 3.** Trial design tentative modifications post-formative work

Prior plan	Post-formative study plan
Give women two HIV self-test kits to take home	Give women three HIV self-test kits to take home^a^
Give voucher incentives	Give cash incentives
Have a medium incentive intervention arm	No longer have medium incentive arm
Give US$15 in the high incentive arm	Give US$10 in the high incentive arm
Only one winner in the lottery incentive arm	No modification to allow quantitative comparison
Send SMS to participants in the reminder arm	Make phone call in the reminder arm

^a^No change in the final design due to budgetary constraints.

High acceptability rates of HIVST have been documented globally in the general population [26], healthcare workers [27], key populations particularly among men who have sex with men [28] and people coming to outpatient departments [29]. The findings observed here indicate very high perceived acceptability, and these results are consistent with results from quantitative studies within the African region [23]. Two recent studies in Kenya showed that the uptake of HIV testing among male partners was 2–3 times higher in the arm that provided two self-test kits to pregnant women attending ANC compared to a standard male partner invitation letter [13,14]. However, the uptake of HIV testing was measured through surrogate reporting by the women or through self-reporting by men in both studies, which may in either case have led to overestimation. The ANC set-up was clearly not conducive for couple testing as many of our participants expressed concerns around stigma, discrimination and lack of privacy, barriers which are directly addressed by home use of HIVST kits. These and other well-known barriers imply that currently strategies may not achieve high uptake among men and couples [16,18,30].

Our data show that physical IPV was perceived to be less likely to occur for pregnant women delivering self-test kits to the partner especially if the kit were introduced at the right moment such as bedtime. This finding contrasts sharply with literature which suggests high prevalence of IPV among pregnant women within the African region (around 15%) [31] and in Malawi (21%) [32]. However, in relationships with pre-existing IPV, participants suggested a more careful approach when introducing the topic of HIVST with bedtime suggested as the best time. It is important to also note that this perception that physical IPV may not occur may not generalize to other settings and may not apply to all forms of IPV such as controlling behaviours or psychological, verbal and economic abuse. Two cases of IPV were reported among postpartum women in a woman-delivered model in Kenya [13]. In both cases, partners reconciled and in one case a man who was HIV-positive started HIV care. This study from Kenya used woman’s reporting of male partner testing as the primary outcome whereas our design is around the proportion of men who test and link to the clinic.

Our findings that deliberate additional interventions to encourage male partners who self-test to link into care or prevention services are needed are consistent with an earlier controlled randomized trial conducted in Malawi that reported that the offer of optional home initiation of two weeks’ worth of ART increased demand for ART compared to providing self-test kits only [33]. Furthermore, participants in the study cited well-documented barriers that may pose additional challenges for people to link into ART or HIV prevention following HIVST [,^34^]. A notable intrinsic barrier with HIVST is that individuals are diagnosed early in their disease progression implying that they may be less likely to prioritize the linkage step, and this may be particularly difficult for men [30].

The data presented here suggest that the enduring economic pressure including finding food for the day that male partners face especially during the time their partner is pregnant may exacerbate their reluctance to seek HIV care or let alone prevention services [35]. Therefore, offering a cash financial incentive conditional on clinic attendance and receiving HIV care or prevention services may enable men to compensate for opportunity cost associated with clinic attendance. Views expressed here suggest that there is need to carefully design the type of financial incentive-based interventions as lottery type of incentives were considered as likely to be less effective than fixed incentives. Two recent randomized controlled trials, one in Kenya [36] and another in Tanzania [37], showed no significant difference in the uptake of voluntary medical male circumcision between a lottery-based intervention and a control arm. These quantitative studies render more weight to our findings which suggest that in settings with extreme poverty, lotteries are perceived as potentially counter-effective. Whilst we are uncertain why this may be the case, a possible explanation is that lotteries may take one’s only hope of winning a big prize away. Furthermore, these findings may be explained by contextual differences or indeed the way lottery was presented to study participants.

Providing reminders is a low-cost intervention that was thought of as an important strategy to ensure that male partners remember to prioritize a clinic appointment over other daily pressing activities. However, our results showed that an SMS reminder is not perceived as a good strategy for improving linkage. Based on other studies, the SMS intervention has shown effect in increasing HIV testing [38] and improving ART adherence [39] although other studies have reported no discernible difference for viral suppression [40] and adherence to TB treatment [41]. We found two aspects of the proposed SMS intervention to be potentially problematic and hence likely to lead to no effect: first, participants may not be able to actually read the contents of the SMS with the influx of “junk” SMSs due to unsolicited adverts leading to participants ignoring or deleting the SMS when it is just received. Second, due to the monologue nature of the SMS, participants may have unanswered questions relating to how to find the clinic or aspects of study procedures. Therefore, a phone call was suggested by the participants as the best form of reminder over an SMS.

Following this formative study, a number of modifications were made to the initial design of the trial (Table 3) including giving cash as opposed to voucher incentives; reducing the high incentive amount from $15 to $10; and making a phone call as opposed to sending an SMS as a reminder. Due to infidelity, some men expressed preference to self-test alone first before repeat couple self-testing. This implied that three self-test kits should be provided to the woman during her ANC visit. The project budget does not allow us to provide three self-test kits as preferred by the participants. Similarly, a decision was made to maintain the lottery arm in the trial in order to formerly test the effect of a lottery incentive on linkage into ART or HIV prevention in this population.

We acknowledge a number of potential limitations. First, being a qualitative study means that we were unable to actually offer participants the choices or the interventions in order to measure uptake of testing and linkage into care or prevention. Second, participants were purposively sampled such that the views may not necessarily represent all pregnant women and their male partners. The final set of interventions would have been best critiqued through a stakeholder workshop before formal testing in the trial but we were unable to conduct the workshop due to lack of time. However, high participation was observed in the FGDs even for mixed gender FGDs. The additional IDIs with previous FGD participants strengthen our study findings further as participants in the IDIs were now better placed to give their individual account having understood the questions from the FGD. Finally, we did not know the HIV status of the study participants as clearly this would shape one’s views differently. However, as we recruited women after receipt of ANC service, we believe they knew their HIV status at the time of the FGD or IDI.

### Conclusions

Woman-delivered HIVST was perceived as highly acceptable to both male partners and their pregnant women attending ANC in urban Blantyre, Malawi. The introduction of this model was not considered to lead to adverse events including IPV. However, additional interventions will likely be required to encourage male partners who self-test to link into HIV care and HIV prevention including the use of conditional financial incentives. Feedback from the study was used to alter the design of a multi-arm multi-stage cluster-randomized trial.
